# The enigma of a subluxated globe

**DOI:** 10.3205/oc000236

**Published:** 2024-04-19

**Authors:** Pratheeba Devi Nivean, T. S. Mohammed Sayee, Sonam Nisar, Nivean Madhivanan

**Affiliations:** 1 MN Eye Hospital, Chennai, India; 2 Arunai Medical College and Hospital, Vellore, India; 3 Medical Research Foundation, Chennai, India

**Keywords:** spontaneous globe subluxation, lateral tarsorrhaphy, medical emergency, shallow orbits

## Abstract

Spontaneous globe subluxation (SGS) is an uncommon condition wherein the equator of the globe protrudes anteriorly beyond the eyelid aperture causing severe lagophthalmos, proptosis and exposure keratopathy. SGS can lead to an emotional disturbance leading to anxiety and fear, thereby affecting one’s quality of life. The patients might often be able to reduce the globe on their own, but permanent measures must be taken to prevent recurrence and vision-threatening sequelae of SGS. We present this case due to its rarity and to highlight the importance of a simple, cost-effective and cosmetically acceptable bilateral tarsorrhaphy in management of SGS.

## Introduction

Spontaneous globe subluxation (SGS) is an uncommon ocular emergency, wherein the equator of the globe protrudes anteriorly beyond the eyelid aperture. Further contraction of the orbicularis oculi causes eyelid retraction and further anterior protrusion of the globe which can lead to vision-threatening complications like exposure keratitis, optic neuropathy and retinal venous congestion [1]. Immediate management includes manual reduction of the globe, while permanent measures require surgical intervention in recurrent cases to prevent its sequelae.

## Case description

**Figure 1:**
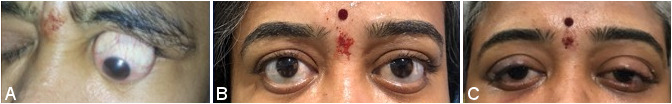
External photograph of the patient with globe subluxation (A); external photograph of the patient at 1-week review after reduction (B); postoperative picture of the patient (C)

A 44-year-old anxious female patient presented to the ophthalmic emergency department with complaints of acute prominence of the left eye associated with pain and diminution of vision. She had two similar episodes in a span of 3 weeks (Figure 1A (Fig. 1)). She was obese and a known hypothyroid patient. On examination, best-corrected visual acuity (BCVA) was 6/9 and 6/6 respectively. Pupils were normal size reacting to light. There was gross left proptosis with the upper and lower lid retracted behind the globe. Left conjunctival hyperemia with exposure keratopathy changes was seen. Diagnosis of a spontaneous globe subluxation was made based on history and clinical examination. An immediate quick maneuver to reduce and reposit the globe was done under topical anaesthesia.

With patient looking downwards, the left upper lid was held firmly and lifted upward off the globe downward and backward. Gentle pressure was given with the index finger on the upper sclera to push the globe back to its place. While doing so, the upper lid was brought anterior to the equator of the globe. Once the globe was repositioned back into the orbit, prophylactic topical antibiotics and lubricants were prescribed.

On subsequent follow-up after 2 days, the patient was comfortable (Figure 1B (Fig. 1)) and her anterior segment and fundus examination were within normal limits in both eyes. Humphrey’s visual field evaluation was within normal limits. Magnetic resonance imaging (MRI) orbit showed bilateral shallow orbits. Considering the recurrent nature of the disease, bilateral lateral tarsorrhaphy was done.

## Procedure

Under local infiltration anaesthesia with 2% lignocaine, the desired area of lateral tarsorrhaphy was assessed and marked bilaterally. The incision was made at the grey line using 11 number blade and 1 mm of the posterior lamellae was excised. The raw ends of both upper and lower lid posterior lamella were approximated with absorbable vicryl 6–0 sutures and anterior lamella (skin) approximated over the posterior lamella with non-absorbable sutures with a tie over bolster to avoid cheese wiring. Suture removal was done after 10 days (Figure 1C (Fig. 1)). At a follow-up of 6 months post-surgery, the tarsorrhaphy was holding on well. The patient was asymptomatic without any further episodes of SGS. 

## Discussion

Risk factors for SGS include obesity, thyroid eye disease, floppy eyelid syndrome, shallow orbits, malar hypoplasia and craniosynostosis. SGS can be attributed to laxity of the orbital septum and lax canthal tendons along with shallow orbit. The triggering factors for globe subluxation can be any eyelid manipulations (rubbing), contact lens use and raised valsalva [1]. Rapid weight gain, shallow orbits and obesity have been associated with SGS. Weight loss in such patients may prevent further episodes of SGS [2], [3], [4]. 

Vivek Gupta et al. have published the normal orbital dimensions amongst the Indian population [2]. They report that almost one third of the globe should fall below the inter-zygomatic line and if the distance reduces along with a reduced depth of orbit then it is considered as a shallow orbit.

Álvaro Bengoa-González et al. in their case series including 13 patients performed a deep, lateral rim-sparing orbital decompression in patients with shallow orbits and reported a successful outcome with no further recurrence [1]. Orbital decompression provides a natural retroplacement of the globe back into the orbit. However, orbital decompression is a complex surgery and a skilled oculoplastic surgeon to perform is needed to perform it. If the patient has associated floppy eyelids or lid laxity, it can trigger further subluxations even if the globe is placed posteriorly. Hence, eyelid laxity must be also addressed.

Our patient had recurrent SGS in only one eye. Imaging revealed bilateral shallow orbits with inactive thyroid eye disease. Despite having a unilateral presentation we performed a bilateral permanent lateral tarsorrhaphy. This was not only aesthetically superior to a unilateral intervention but also prevented any potential episode of SGS in the other eye. 

Permanent lateral tarsorrhaphy is a simple, cost- and time-effective procedure which can be easily done by a general ophthalmologist. 

A reduction in the horizontal palpebral fissure takes care of the lid laxity and also prevents further globe subluxation. Lateral tarsorrhaphy has been reported to produce a tighter orbit and increase the intraorbital pressure. The smaller remaining palpebral aperture can cause further difficulty in repositioning the globe in case of a recurrence [5]. However, in our case at 6 months follow-up, the patient was comfortable without any repeated episodes of globe subluxation.

Hari Mylvaganam et al. have reported increased venous congestion and decreased venous return in cases of globe subluxation. This fluid collection can often mimic an orbital abscess on imaging [6]. One can use a paediatric desemarres retractor or an easily available large paper clip to mobilise the lid before globe reposition in case of difficulty [7].

## Conclusion

Spontaneous globe subluxation though uncommon can cause undue apprehension and panic amongst patients. General ophthalmologists must be well aware of this condition and the globe reduction techniques in the acute phase. A timely surgical intervention in recurrent cases prevents further morbidity.

## Notes

### Competing interests

The authors declare that they have no competing interests.
